# Occurrence of *Hepatozoon canis* (Adeleorina: Hepatozoidae) and *Anaplasma* spp. (Rickettsiales: Anaplasmataceae) in black-backed jackals (*Canis mesomelas*) in South Africa

**DOI:** 10.1186/s13071-018-2714-y

**Published:** 2018-03-20

**Authors:** Barend L. Penzhorn, Edward C. Netherlands, Courtney A. Cook, Nico J. Smit, Ilse Vorster, Robert F. Harrison-White, Marinda C. Oosthuizen

**Affiliations:** 1 Vectors and Vector-borne Diseases Research Programme, Department of Veterinary Tropical Diseases, Faculty of Veterinary Science, University of Pretoria, Private Bag X04, Onderstepoort, 0110 South Africa; 2 National Zoological Gardens, Boom Street, Pretoria, South Africa; 3 Water Research Group, Unit for Environmental Sciences and Management, North-West University, Private Bag X6001, Potchefstroom, 2520 South Africa; 4 African Amphibian Conservation Research Group, Unit for Environmental Sciences and Management, North-West University, Private Bag X6001, Potchefstroom, 2520 South Africa; 5 Laboratory of Aquatic Ecology, Evolution and Conservation, University of Leuven, Charles Debériotstraat 32, B-3000 Leuven, Belgium; 6 Department of Zoology and Entomology, University of the Free State, Qwaqwa, South Africa; 7 Wildlife Damage - Research and Management, P.O. Box 783540, Sandton, 2146 South Africa

**Keywords:** *Anaplasma*, Black-backed jackal, *Canis mesomelas*, *Hepatozoon canis*, South Africa

## Abstract

**Background:**

Domestic dogs are not native to sub-Saharan Africa, which may account for their susceptibility to *Babesia rossi*, of which endemic black-backed jackals (*Canis mesomelas*) are natural reservoirs. There is virtually no information on the occurrence of potentially pathogenic haemogregarines (e.g. *Hepatozoon canis*) or even rickettsial bacteria (e.g. *Ehrlichia* spp. and *Anaplasma* spp.) in indigenous canids in sub-Saharan Africa. Such organisms could pose a risk to domestic dogs, as well as to populations of endangered indigenous canid species.

**Results:**

Genomic DNA extracted from blood samples taken from 126 free-ranging and 16 captive black-backed jackals was subjected to reverse line blot (RLB) hybridization assay; 82 (57.8%) specimens reacted only with the *Ehrlichia*/*Anaplasma* genera-specific probe. Full-length bacterial *16S* rRNA gene of five of these specimens was cloned and the recombinants sequenced. The ten *16S* rDNA sequences obtained were most closely related, with approximately 99% identity, to *Anaplasma* sp. South African Dog, various uncultured *Anaplasma* spp., as well as various *Anaplasma phagocytophilum* genotypes. Ninety-one specimens were screened for haemogregarines through PCR amplification using the *18S* rRNA gene; 20 (21.9%) specimens reacted positively, of which 14 (15.4%) were confirmed positive for *Hepatozoon* genotypes from within *H. canis*. Two (2.2%) specimens were found positive for two different *Hepatozoon* genotypes.

**Conclusions:**

Sequence analyses confirmed the presence of *16S* rDNA sequences closely related to *A. phagocytophilum* and *Anaplasma* sp. South African Dog as well as two *H. canis* genotypes in both free-ranging and captive black-backed jackals. Distinguishing between closely related lineages may provide insight into differences in pathogenicity and virulence of various *Anaplasma* and *H. canis* genotypes. By building up a more comprehensive understanding of the range and diversity of the bacteria and eukaryotic organisms (piroplasms and haemogregarines) in the blood of indigenous canids, we may gain insight to such infections in these often-endangered species and the potential for horizontal transmission to and from domestic dogs via ticks where favourable conditions exist.

## Background

Domestic dogs are not native to sub-Saharan Africa [1]. This may account for their susceptibility, especially recently introduced pure-bred dogs, to pathogens harboured by endemic indigenous canids [2]. Black-backed jackals (*Canis mesomelas*) have recently been shown to be natural reservoirs of *Babesia rossi* which causes a potentially fatal disease in domestic dogs [3], but it is not known whether the same applies to other potentially pathogenic apicomplexan protozoa (e.g. *Hepatozoon canis*) and rickettsial bacteria (e.g. *Ehrlichia* spp. and *Anaplasma* spp.). Knowledge about the occurrence of such organisms in indigenous canids such as jackals and African wild dogs (*Lycaon pictus*) is important to assess the risk that indigenous canid species could pose as reservoirs of pathogens that could be transmitted to domestic dogs. Conversely, domestic dogs could serve as reservoirs of infection with pathogens that could negatively affect populations of rare or endangered indigenous canids.

Apart from black-backed jackals, sub-Saharan Africa hosts three indigenous *Canis* species, i.e. the side-striped jackal (*C. adustus*), the African golden wolf (*C. anthus*) and the Ethiopian wolf (*C. simensis*). Of these, side-striped jackals have the widest distribution, followed by black-backed jackals which occur in two discrete geographic ranges, separated by 900 km: Northeast Africa and Southwestern Africa [4, 5]. African golden wolves occur from northern Tanzania northwards and westwards through the Sahelian region [6]. Ethiopian wolves, an endangered species, occur only in the highlands of Ethiopia [7]. Previously widespread, African wild dogs have disappeared from most of their historic range due to on-going habitat fragmentation, livestock ranching and infectious disease; they are also regarded as endangered [8].

*Anaplasma phagocytophilum*, an emerging pathogen of humans, horses and dogs worldwide, was recently reported from South Africa [9]. A closely related but distinct species, referred to as *Anaplasma* sp. South African Dog, had previously been reported from domestic dogs in South Africa [10]. Since *A. phagocytophilum* has zoonotic potential, it would be important to determine whether the widespread black-backed jackals also harbour these infections.

It has recently been demonstrated that there is marked genetic diversity in *Hepatozoon* spp. in coyotes (*Canis latrans*) in the USA [11]. The same may therefore apply in canid populations elsewhere. *Hepatozoon* spp. have occasionally been identified in African canids [12–14]; whether this was *H. canis* is a moot point, since identification was not based on molecular characterisation, but on morphology.

Black-backed jackals are known to host *Hepatozoon* spp. Significant lesions attributed to hepatozoonosis were described in three black-backed jackals from Kruger National Park, South Africa [12]. Schizonts were found in skeletal muscles, lungs and bone marrow, with the diaphragm, muscles of the limbs and pectoral muscles being most heavily parasitized. Although focal, the accompanying myositis was severe, with necrosis of individual cells [12].

A project aimed at developing ecologically friendly strategies for managing problem carnivores on farmland in South Africa offered an opportunity to collect a large set of blood specimens from free-ranging black-backed jackals [3]. Specimens taken routinely whenever jackals were handled, e.g. for fitting radio collars, were submitted to the Department of Veterinary Tropical Diseases (DVTD), University of Pretoria (UP) to determine the occurrence of haemoprotozoa and rickettsial bacteria [3].

## Methods

### Sample collection

Free-ranging black-backed jackals (*n* = 126) at Mogale’s Gate Biodiversity Centre (25°55'51"S, 27°38'33"E) at the border between North West Province and Gauteng Province, South Africa, were immobilised by intramuscular injection of a combination of tiletamine and zolazepam (Zoletil®, Virbac RSA, Centurion, South Africa). Blood specimens collected into EDTA tubes from the cephalic vein were frozen and submitted to the Molecular Biology Laboratory, DVTD, UP. For comparative purposes, blood specimens were collected from captive black-backed jackals (*n* = 16) at S.A. Lombard Nature Reserve (27°37'35"S, 25°34'51"E), North West Province, South Africa.

### DNA extraction

To determine presence of *Anaplasma* spp. and/or *Ehrlichia* spp., genomic DNA was extracted at the DVTD, UP, from the EDTA blood samples (*n* = 142) using the QIAamp® DNA Mini Kit (Qiagen, Southern Cross Biotechnologies, Cape Town, South Africa) according to the manufacturer’s instructions. DNA was eluted in 100 μl elution buffer and stored at -20 °C. To determine the presence of haemogregarines a subset of blood samples (*n* = 91) was submitted to the Unit for Environmental Sciences and Management, North-West University, Potchefstroom, South Africa, where genomic DNA was extracted using the KAPA Express Extract Kit (Kapa Biosystems, Cape Town, South Africa).

### Reverse line blot (RLB) hybridisation

The RLB hybridisation assay was done according to Gubbels et al. [15] and Nagore et al. [16]. The V1 hypervariable region of the bacterial *16S* rRNA gene was amplified using primers Ehr-F (5'-GGA ATT CAG AGT TGG ATC MTG GYT CAG-3') [17] and Ehr-R (5'-Biotin-CGG GAT CCC GAG TTT GCC GGG ACT TYT TCT-3') [17]. The touchdown PCR thermocycler program, as described by Nijhof et al. [18], was used to perform the DNA amplification. *Anaplasma centrale* DNA extracted from a commercial bovine anaplasmosis vaccine (Onderstepoort Biological Products, Tshwane, South Africa) was used as a positive control; the negative control was water. The PCR products were subjected to RLB hybridization as described by Nijhof et al. [18] using *Anaplasma* and *Ehrlichia* genera- and species-specific oligonucleotide probes at predetermined concentrations, including *Anaplasma bovis* [19], *A. centrale* [19], *Anaplasma marginale* [19], *Anaplasma phagocytophilum* [19], *Anaplasma* sp. Omatjenne [19], *Ehrlichia canis* [17], *Ehrlichia chaffeensis* [17] and *Ehrlichia ruminantium* [17]. An *Anaplasma platys* probe (A.M. Nijhof, unpublished observations) was added to the membrane before the last 35 specimens, all from free-ranging jackals, were tested.

### *16S* amplification, cloning, sequencing and phylogenetic analysis

The full-length *16S* rRNA gene of five of the jackal specimens that reacted with the *Ehrlichia*/*Anaplasma* genera-specific probe only was amplified using universal primers fD1 (5'- AGA GTT TGA TCC TGG CTC AG-3') and rP2 (5'-ACG GCT ACC TTG TTA CGA CTT-3') [20]. Five separate reactions were prepared per sample, pooled (to avoid *Taq* polymerase-induced errors) and cleaned-up using the QIAquick PCR Purification Kit (Qiagen). *Anaplasma centrale-*positive DNA and water were used as positive and negative controls, respectively, for the PCR amplification.

Using the CloneJET PCR Cloning Kit (Thermo Fisher Scientific, Waltham, MA, USA), the purified PCR fragment was ligated into the CloneJET vector and transformed into competent *Escherichia coli* JM109 cells (JM109 High Efficiency Competent Cells, Promega, Madison, WI, USA). Recombinant plasmids were isolated using the High Pure Plasmid Isolation Kit (Roche Diagnostics, Mannheim, Germany). Sequencing was performed at InqabaBiotec™ (Pretoria, South Africa).

The obtained sequences were assembled and edited using the GAP4 program of the Staden package (version 1.6.0 for Windows) [21]. A BLASTn homology search [22] of GenBank was done using the full length consensus sequences. These were then aligned with *16S* rRNA gene sequences of related genera using ClustalX (version 1.81 for Windows) [23]. The alignments were manually examined and then truncated to the size of the smallest sequence (1323 bp) using BioEdit version 7 [24]. Ten *16S* rRNA gene sequences were analysed. Estimated evolutionary divergence was calculated by determining the number of nucleotide differences between similar sequences. All positions containing gaps and missing data were eliminated. There was a total of 1318 positions in the final dataset.

### *18S* rRNA gene amplification, cloning and sequencing

Once extracted, DNA was used for PCR amplification. Following the methods of Cook et al. [25], identification of haemogregarines was initially completed using the primer set HepF300 (5'-GTT TCT GAC CTA TCA GCT TTC GAC G-3') and HepR900 (5'-CAA ATC TAA GAA TTT CAC CTC TGA C-3'). The PCR reactions were run targeting a fragment (approximately 600 bp) of the *18S* rRNA gene [26]. A second PCR was carried out using the primer set 4558 (5'-GCT AAT ACA TGA GCA AAA TCT CAA-3') and 2733 (5'-CGG AAT TAA CCA GAC AAA T-3') [27], targeting a fragment (approximately 1120 bp) of the *18S* rRNA gene. PCR reactions were performed with volumes of 25 μl, using 12.5 μl Thermo Scientific DreamTaq PCR master mix (2×) (final concentration: 2× DreamTaq buffer, 0.4 mM of each dNTP, and 4 mM MgCl2), 1.25 μl (10 μM) of each of the primer sets mentioned above, and at least 25 ng DNA. The final reaction volume was made up with PCR-grade nuclease-free water (Thermo Scientific). Reactions were undertaken in a Bio-Rad C1000 Touch™ Thermal Cycler PCR machine (Bio-Rad, Hemel Hempstead, UK). PCR conditions were as follows: initial denaturation at 94 °C for 3 min, followed by 40 cycles, entailing a 94 °C denaturation for 1 min, annealing at 55 °C for 2 min with an end extension at 72 °C for 2 min, and following the cycles a final extension of 72 °C for 10 min [25]. Resulting amplicons were visualised under UV on a 1% agarose gel stained with gel red. PCR products from each sample were sent to a commercial sequencing company (InqabaBiotec™) for purification and sequencing in both directions. Resultant sequences were assembled using Geneious R9.1 (http://www.geneious.com) [28] and chromatogram-based contigs were generated, trimmed and manually corrected for ambiguous base calls. Sequences were identified using the Basic Local Alignment Search Tool (BLAST) [22].

Comparative sequences of *Hemolivia*, *Hepatozoon* and *Haemogregarina* spp. parasitising reptiles, amphibians, mammals and ticks were downloaded from GenBank and aligned to the sequences generated within this study. *Babesiosoma stableri* (GenBank: HQ224961) and *Dactylosoma ranarum* (GenBank: HQ224957) were chosen as outgroup, as in Netherlands et al. [29]. Sequences were aligned using the ClustalW alignment tool [30]. The alignment (553 bp) consisted of 32 sequences. A model test was performed to determine the most suitable nucleotide substitution model, according to the Akaike information criterion using jModelTest version 2.1.7 [31, 32]. The model with the best AICc score was the Transitional model [33] with estimates of invariable sites and a discrete Gamma distribution (TVM + I + Γ). However, this model was substituted by the General Time Reversible model with estimates of invariable sites and a discrete Gamma distribution (GTR + I + Γ) in RAxML [34], as this was the next model available with the best AICc score. To infer phylogenetic relationships, maximum likelihood (ML) analysis was performed using RAxML version 7.2.8. [35], implemented in Geneious R9.1. Nodal support was undertaken with 1000 bootstrap replicates. Only nodal support greater than 70% is shown.

### Statistical analysis

The Chi-square test was performed utilising an open-access online calculator (http://www.socscistatistics.com/tests/chisquare/).

## Results

### *Anaplasma* and/or *Ehrlichia* spp.

On RLB none of the specimens reacted with any species-specific probe; 82 (57.7%) specimens reacted only with the *Anaplasma*/*Ehrlichia* genera-specific probe, which could suggest the presence of a novel species or variant of a species. Eleven (68.8%) of the 16 specimens from captive jackals reacted positively, while 71 (56.3%) of the 126 specimens from free-ranging jackals reacted positively. The difference was not significant (*χ*^2^ = 0.8949, *df* = 1, *P* = 0.344187).

Nine of the ten *16S* rDNA sequences obtained (originating from five jackals) were identical (over 1323 bp); the other sequence (RE17/019/3), obtained from a free-ranging jackal, differed by 1 bp. BLASTn homology search results revealed no identical sequences in the public databases. The most closely related sequences, with approximately 99% identity, were *Anaplasma* sp. South African Dog (GenBank: AY570539 and AY570538), various uncultured *Anaplasma* spp., as well as various *A. phagocytophilum* genotypes.

### *Hepatozoon* spp.

**Fig. 1:**
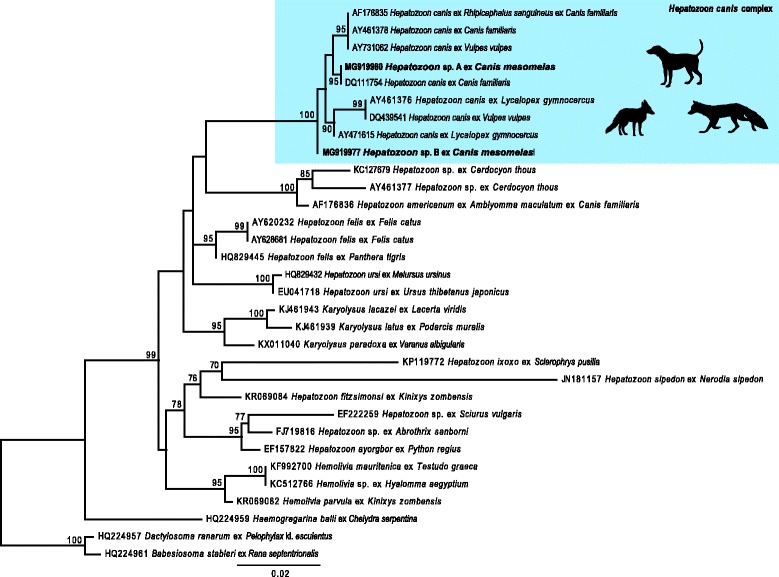
A maximum likelihood tree based on *18S* rDNA nucleotide sequences showing phylogenetic relationships between the apicomplexans. The evolutionary distances were computed using the GTR + I + Γ model. All positions containing gaps and missing data were eliminated. There was a total of 969 positions in the final dataset

Ninety-one blood samples were screened for haemogregarines through PCR amplification. Twenty samples (21.9%) reacted positively, from which 14 (15.4%) sequences were successfully generated. All 14 were positive for a genotype of *Hepatozoon* designated here as *Hepatozoon* genotype A. Of these, two were mixed infections of *Hepatozoon* genotype A and a second genotype designated here as *Hepatozoon* genotype B (Fig. 1). BLAST results of the *18S* rDNA sequence fragments (1024 bp) revealed a 99% identity to *H. canis* (GenBank: DQ111754).

*Hepatozoon* genotypes from this study are well nested within *H. canis*. Furthermore, these sequences fall separate from other *Hepatozoon* spp. clusters, isolated from amphibian, reptile and small mammal/rodent hosts, and the *Hepatozoon americanum*, *Hepatozoon ursi*, and *Hepatozoon felis* clusters, respectively (Fig. 1).

## Discussion

Domestic dogs are absent from Mogale’s Gate Biodiversity Centre, our main study site, but black-backed jackals move freely between this conservancy and surrounding farming areas where domestic dogs are kept. Therefore, tick transfer of infectious agents between domestic dogs and jackals cannot be ruled out.

*Anaplasma phagocytophilum*, which poses a known human-health risk, was recently reported from a dog in South Africa [9]. It is known to occur in golden jackals (*Canis aureus*) in Israel [36]. Although the occurrence of *A. phagocytophilum* was not confirmed in black-backed jackals, the presence of closely related organisms may be cause for concern. *Anaplasma* sp. South African Dog has been recovered from domestic dogs and ticks that had engorged on dogs in various provinces of South Africa [9, 37]. It is imperative, therefore, that the relationship between the various organisms should be elucidated.

None of our specimens reacted with the *E. canis* species-specific probe. This is in striking contrast to a report from Kenya, where black-backed jackals were regarded as a potential reservoir host for *E. canis* [38]. Eight of 16 jackals examined in that study were positive for *E. canis* on a modified cell culture test [38]. In a subsequent study in Kenya, however, only one of 36 black-backed jackals was seropositive to *E. canis* [39]. In South Africa a black-backed jackal exposed to infected ticks contracted fatal ehrlichiosis [40]. After intravenous transmission of blood from infected dogs, four jackals showed no clinical signs but became subclinically infected with *E. canis* [41, 42]. One of these jackals remained infected for at least 112 days [41].

*Rhipicephalus sanguineus* (*sensu lato*) [43] is the only proven vector of *E. canis* [44, 45]. Price et al. [38], who reported a 50% prevalence of *E. canis* in black-backed jackals in Kenya, also reported five of 12 jackals to be infested with *R. sanguineus* (*s.l.*). This record was overlooked or rejected by Walker et al. [46], who did not list jackals as hosts of *R. sanguineus*. This tick species was also not recovered from African wild dogs (*n* = 29) in the Kruger National Park, South Africa, which were all seronegative to *E. canis* [14].

Our results are the first confirmation of the occurrence of *H. canis* in black-backed jackals. In a study conducted in northern Africa, the overall prevalence of *Hepatozoon* spp. was higher in foxes (*Vulpes* spp.) than in jackals (*Canis* spp.) [47]. The *Hepatozoon* sp. reported from a single Cape fox (*Vulpes chama*) in South Africa had a genetic lineage very similar to that found in foxes (*Vulpes* spp.) in northern Africa [47, 48].

Our phylogenetic analysis shows a close relationship for the two *Hepatozoon* genotypes identified during the current study to *Hepatozoon* genotypes from other hosts of the family Canidae, which were generally regarded as belonging to the *H. canis* group, sister to the *H. americanum* group (Fig. 1). Recent studies on other vertebrate classes using both morphological and molecular techniques have proven useful to distinguish between closely related species of *Hepatozoon* [29, 49, 50].

Being able to distinguish between closely related lineages might provide better insights into the pathogenicity and virulence of *H. canis* genotypes, which is often but not always (depending on the parasitaemia) subclinical in dogs [51, 52]. In contrast to the usually mild *H. canis*, *H. americanum*, which is a more virulent species and can be fatal, may have only recently crossed the species barrier from a wild host to the domestic dog [51, 52]. If *Hepatozoon* spp. which naturally infect wild hosts pose a potential cross-over threat not only to domestic hosts, but also to other wild host species, such as in the case of *H. americanum*, it is important to closely monitor these parasites by screening more taxa and building up a more comprehensive molecular database where needed.

## Conclusions

Sequence analyses confirmed the presence of *16S* rDNA sequences closely related to *A. phagocytophilum* and *Anaplasma* sp. South African Dog in both free-ranging and captive jackals. Since *A. phagocytophilum* poses a threat to human health, this should be further investigated. Sequence analyses also confirmed the presence of two *Hepatozoon* genotypes nestled within *H. canis*. Distinguishing between closely related lineages may provide insight into differences in pathogenicity and virulence of various *H. canis* genotypes. Such genotypes naturally infecting wild canids may pose a potential cross-over threat to domestic dogs and other wild hosts, as possibly occurred with *H. americanum.* By building up a more comprehensive understanding of the range and diversity of the bacteria and eukaryotic organisms (piroplasms and haemogregarines) in the blood of indigenous canids, we may gain insight to such infections in these often-endangered species and the potential for horizontal transmission to and from domestic dogs via ticks where favourable conditions exist.

## Abbreviations

DAFF: Department of Agriculture, Forestry and Fisheries, South Africa
DVTD, UP: Department of Veterinary Tropical Diseases, University of Pretoria
EDTA: Ethylenediaminetetraacetic acid
PCR: Polymerase chain reaction
RLB: Reverse line blot
